# The effect of the Apollo Neuro device on anxiety among participants who underwent ketamine assisted therapy: A non-randomized, observational cohort study

**DOI:** 10.1192/j.eurpsy.2026.11223

**Published:** 2026-07-31

**Authors:** S. Wong, V. Tsang, B. Tao, R. Olthuis, P. Allard, S. Bhanot, P. Kyrskow

**Affiliations:** 1 Faculty of Medicine; 2 Department of Psychiatry, University of British Columbia, Vancouver; 3 Roots to Thrive Psychedelic Society, Victoria; 4 Faculty of Medicine, University of Toronto, Toronto, Canada

## Abstract

**Introduction:**

Positive feedback from a mental health treatment program that uses treatment adjuncts inspired researchers to assess the potential role of the Apollo Neuro device in mental health outcomes.

**Objectives:**

To assess the impact of the Apollo Neuro device on anxiety levels in a ketamine-assisted therapy treatment program, comparing those using the device with those not using the device.

**Methods:**

This is a quantitative study where the primary outcome measure was the change in Generalized Anxiety Disorder 7-item (GAD-7) scores, calculated as the difference between pre-GAD-7 and post-GAD-7 scores between patients with and without the Apollo Neuro device. Data cleaning was performed to handle missing values and non-numeric entries. Propensity score matching was used to match participants from the “Apollo” and “Control” groups based on demographic and baseline GAD-7 scores. An independent sample t-test was conducted to compare the mean change in GAD-7 scores between the two groups.

**Results:**

Shapiro test did not suggest non-normality of GAD-7 change over the duration of the treatment program. Without pre-balancing of age/sex/pre-gad-7, an independent Welch Two Sample t-test was conducted to compare the mean change in GAD-7 scores between the two groups. There was no significant difference (p=.57).

**Conclusions:**

Despite limitations of randomization and diversified participant groups, GAD-7 score analysis revealed median reduction in anxiety symptoms both with and without the Apollo Neuro device. Future research should further explore the effectiveness of the Apollo Neuro device in reducing anxiety symptoms, ideally through randomized controlled trials with larger and more diverse participant samples.

**Disclosure of Interest:**

None Declared

